# Entomotoxic efficacy of fungus-synthesized nanoparticles against immature stages of stored bean pests

**DOI:** 10.1038/s41598-023-35697-1

**Published:** 2023-05-25

**Authors:** Eman Ahmed Mohamed Helmy, Phyu Phyu San, Yao Zhuo Zhang, Charles Adarkwah, Midori Tuda

**Affiliations:** 1 The Regional Centre for Mycology and Biotechnology (RCMB), Al-Azhar University, Cairo, Egypt; 2 Laboratory of Insect Natural Enemies, Institute of Biological Control, Faculty of Agriculture, Kyushu University, Fukuoka, 819-0395 Japan; 3 Department of Entomology and Zoology, Yezin Agricultural University, Naypyitaw, Myanmar; 4 Department of Horticulture and Crop Production, School of Agriculture and Technology, Dormaa-Ahenkro Campus, University of Energy and Natural Resources, PO Box 214, Sunyani, Ghana; 5 Division Urban Plant Ecophysiology, Faculty Life Sciences, Humboldt-University of Berlin, Lentzeallee 55/57, 14195 Berlin, Germany

**Keywords:** Nanobiotechnology, Model invertebrates, Entomology

## Abstract

Nanopesticides, particularly biosynthesized ones using organic reductants, hold great promise as a cost-effective and eco-friendly alternative to chemical pesticides. However, their efficacy on stored product pests, which can cause damage to dried grains, has not been extensively tested, especially on immature stages. Here, we biosynthesized six types of nanoparticles (NPs) using extracts from the fungus *Fusarium solani*: silver (AgNPs), selenium (SeNPs), silicon dioxide (SiO_2_NPs), copper oxide (CuONPs), titanium dioxide (TiO_2_NPs) and zinc oxide (ZnONPs) ranging in size from 8 to 33 nm. To test their efficacy on stored bean pests, they were applied to the eggs and larvae of pest beetles *Callosobruchus chinensis* and *Callosobruchus maculatus* (Coleoptera: Chrysomelidae: Bruchinae), which burrow into seeds as larvae. Susceptibility to the NPs was species-dependent and differed between developmental stages; eggs were more susceptible than larvae inhabiting in seeds. SeNPs and TiO_2_NPs reduced the hatchability of *C. chinensis* eggs by 23% and 18% compared to the control, respectively, leading to an 18% reduction in egg-to-adult survival by SeNPs. In *C. maculatus*, TiO_2_NPs applied to eggs reduced larva-to-adult survivorship by 11%, resulting in a 15% reduction in egg-to-adult survival. The egg mass of *C. chinensis* was 23% smaller than that of *C. maculatus*: the higher surface-area-to-volume ratio of the *C. chinensis* eggs could explain their higher acute mortality caused by the NPs compared to *C. maculatus* eggs. The biosynthesized SeNPs and TiO_2_NPs have potential for controlling major stored bean pests when applied to their eggs. This is the first to show the efficacy of biosynthesized SeNPs and TiO_2_NPs on stored product pests and the efficacy of *Fusarium*-synthesized NPs on insects.

## Introduction

The world population has reached 8 billion in 2022 and is projected to peak at 10.4 billion by the 2080s^1^. Pulses such as cowpeas (*Vigna unguiculata*), mung beans (*Vigna radiata*), and azuki beans (*Vigna angularis*) are among the most significant protein sources for the human populations of different cultures and vegetarians^2^. However, storage losses caused by insect pests such as *Callosobruchus* beetles (Coleoptera: Chrysomelidae: Bruchinae) can have a significant impact on this important food supply. The cowpea beetle (*C. maculatus*) in tropical areas and azuki bean beetle (*C. chinensis*) in temperate areas are important stored product pests. These pests have wide host ranges^3,4^ and can cause severe losses to a majority of dried beans (up to 20% and occasionally higher^5,6^). Geographical habitat ranges are also expanding^7–9^, making control of these stored product pests crucial in reducing such losses. Furthermore, *Callosobruchus* beetles serve as model organisms for population studies^10,11^.

While chemical insecticides such as fumigants and inert materials such as dusts are effective in controlling bruchine beetles and other stored pests, their use in farmer’s storage facilities, which are often not airtight, can pose risks to human health and the environment^12^. Therefore, researchers are exploring alternative insecticides to protect both agriculture and ecosystems. One promising approach for stored product protection is the use of nanoparticle formulations^13,14^. Nanoparticles (NPs) have unique features, such as a high surface-area-to-volume ratio, high reactivity, and enhanced catalytic and biological properties^15^, making them suitable for a variety of applications, including agriculture^16^.

Metal and metallic oxide NPs such as silver (Ag), zinc oxide (ZnO), copper oxide (CuO), silica (silicon dioxide, SiO_2_), titanium dioxide (TiO_2_), gold (Au), and aluminum oxide (Al_2_O_3_) are being developed for pest and disease control. For instance, SiO_2_NPs have been demonstrated to have physisorption in cuticle lipids of insects, leading to their mortality^17^. SiO_2_NPs have also been found to alter volatile emissions from infested plants, which attracts predators^18^. Selenium nanoparticles (SeNPs) possess antioxidant^19^, antibacterial^20^, anticancer^21^, neuroprotective^22^, antimicrobial^23^, and plant-growth-promoting properties^24^, and can be used in various medical and agricultural treatments^25^. Recent studies have demonstrated the insecticidal effect of SeNPs on a moth and a beetle^14,26^. TiO_2_NPs are used in suncreens and cosmetics to protect from UV and in paint and food coloration. TiO_2_NPs can affect soil invertebrates as well as control insect pests such as moths, coleopterans and hemipterans^27–31^.

Biosynthesized NPs are expected to transform the field of integrated pest management (IPM) in the future^32,33^. Compared to chemical synthesis, the biological synthesis of nanopesticides using plant extracts and microbes is greener, and the produced NPs are stable, environmentally friendly, and cost-effective: They do not require high temperature, high pressure, high energy, or toxic chemicals and do not produce by-products with mammalian toxicity^34–38^. For example, SeNPs can be synthesized using bacteria^23^ and fungi (e.g. *Mariannaea* sp.^39^). Similarly, TiO_2_NPs can be synthesized using bacteria^40^ and plant extracts^41^. Various species of fungi have also shown potential for use in biogenic synthesis of NPs with different characteristics^42^. The fungus *Fusarium* sp. has been used for the extracellular biosynthesis of AgNPs^43^. However, the efficacy of biosynthesized NPs on stored product pest beetles has been studied on a limited number of species (*Sitophilus oryzae*, *Tribolium castaneum*, *Tenebrio molitor*, and *C. maculatus*^44–47^). For example, ZnONPs synthesized with leaf extract or entomopathogens^48,49^ and NiNPs synthesized using plant extracts^44,50^ have been tested on adult *C. maculatus*.

In almost all cases, the targeted developmental stage of the studied stored product pests by NPs has been the adult stage, and the comparison of NP efficacy has rarely been made between developmental stages of pests^44–46,51^. Abdel-Raheem et al.^52^ tested the efficacy of AgNPs synthesized with entomopathogenic fungi on the egg, larva, and adult stages of the red palm weevil *Rhynchophorus ferrugineus*. However, it is not yet known whether the result of this comparison can be applied to immature stages of other coleopterans (weevils and beetles) that have the potential to be exposed to pesticides to different extents. Therefore, in this study, we aimed to test the efficacy of biosynthesized NPs of Ag, CuO, Se, SiO_2_, TiO_2_, and ZnO by *Fusarium solani* extract as insecticides against two *Callosobruchus* beetle species at two immature stages, egg (attached to the surface of seeds) and larva (feeding seeds internally). We hypothesized that the biosynthesized NPs would reduce the survival of both species, regardless of the developmental stage treated. This is the first study to test the control efficacy of *Fusarium*-synthesized NPs on insects, as well as biosynthesized Se, SiO_2_, and TiO_2_ NPs on stored product pests.

## Results

### Control efficacy on *Callosobruchus chinensis*

#### Treatment on eggs of *C. chinensis*

**Figure 1:**
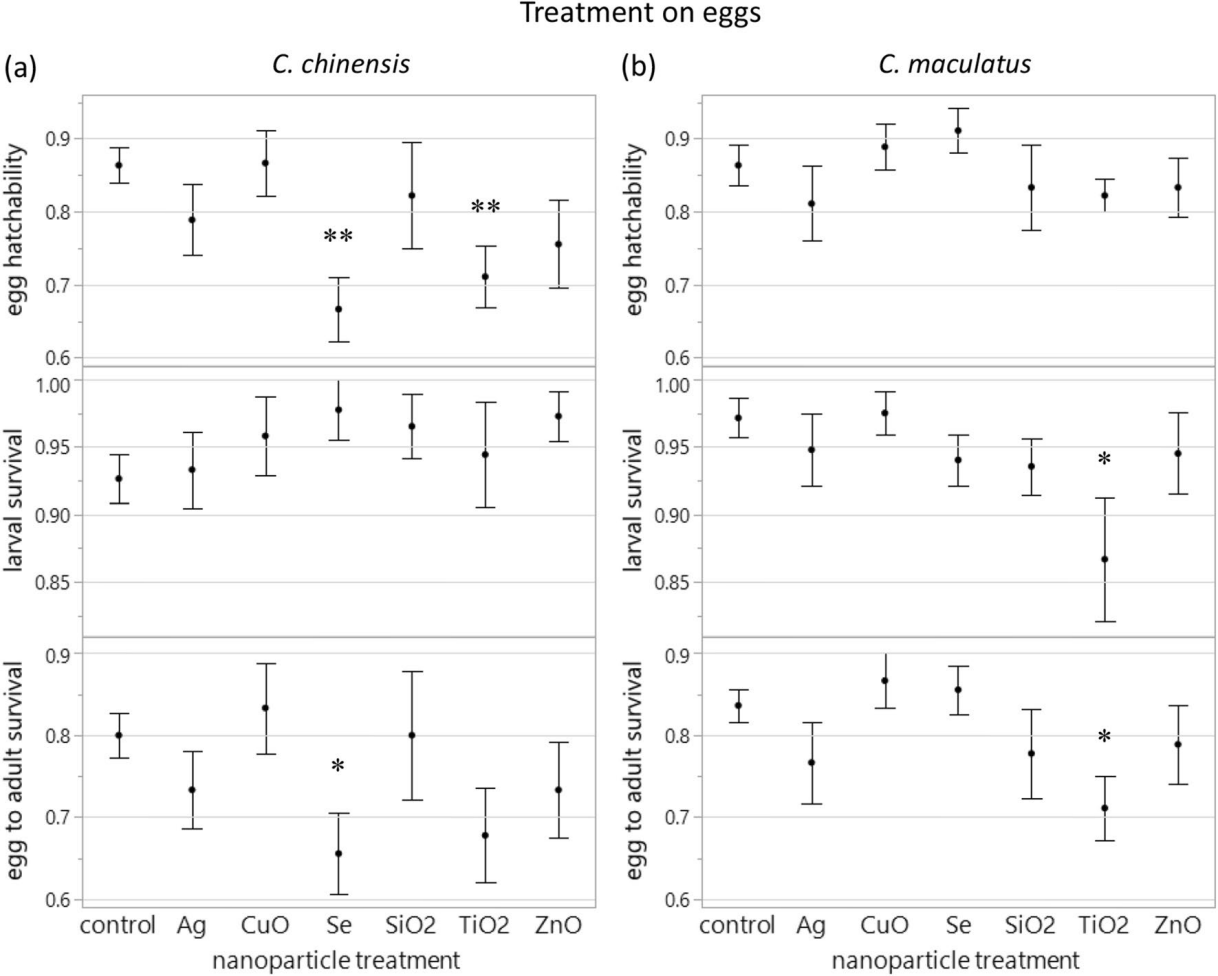
Survival (mean ± SE) of (**a**) *Callosobruchus chinensis* and (**b**) *Callosobruchus maculatus* when eggs were treated with different types of biosynthesized nanoparticles. *P < 0.05, **P < 0.01 compared to the control.

For eggs treated with NPs, there was a significant effect of NP element on hatchability of eggs [LR (likelihood-ratio) *χ*^2^_6_ = 19.09, *P* = 0.004]. Specifically, SeNPs and TiO_2_NPs reduced the egg hatchability by 22.8% and 17.7%, respectively, compared to the control (posthoc comparison with the control, SeNPs, *P* < 0.001; TiO_2_NPs, *P* = 0.008, Fig. 1a). Larva-to-adult survival was not affected by NP element (LR *χ*^2^_6_ = 5.09, *P* = 0.533, Fig. 1a). However, egg-to-adult survival was affected (LR *χ*^2^_6_ = 13.06, *P* = 0.042): SeNPs reduced egg-to-adult survival by 18.1% compared to the control (*P* = 0.021).

#### Treatment on larvae of *C. chinensis*

**Figure 2:**
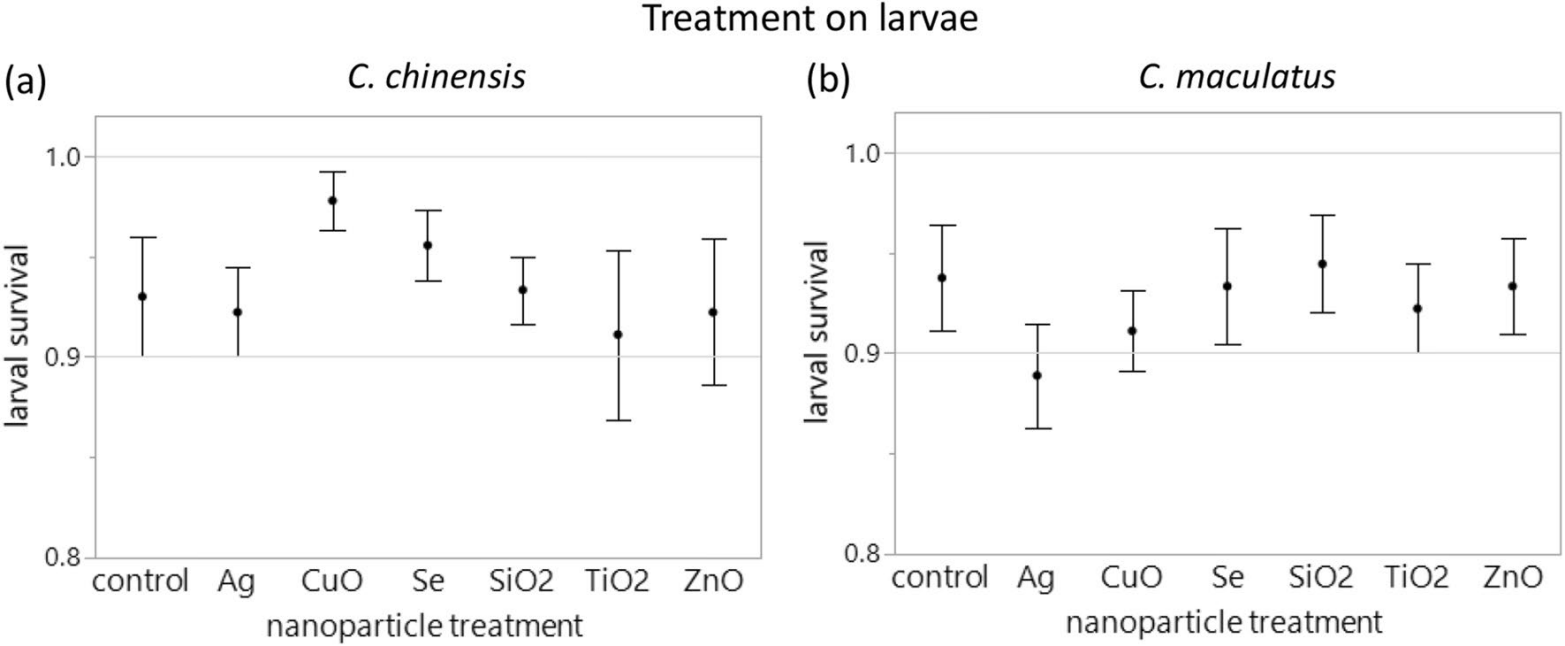
Larva-to-adult survival (mean ± SE) of (**a**) *Callosobruchus chinensis* and (**b**) *Callosobruchus maculatus* when larvae were treated with different types of biosynthesized nanoparticles. No significant difference compared to the control was found in each species.

There was no difference among the NP elements and the control in larva-to-adult survival (LR *χ*^2^_6_ = 5.53, *P* = 0.477, Fig. 2a).

### Control efficacy on *Callosobruchus maculatus*

#### Treatment on eggs of *C. maculatus*

For eggs treated with NPs, there was no significant effect of NP element on hatchability of eggs (LR *χ*^2^_6_ = 6.21, *P* = 0.400), larva-to-adult survival (LR *χ*^2^_6_ = 9.56, *P* = 0.144), egg-to-adult survival (LR *χ*^2^_6_ = 10.56, *P* = 0.103), or the number of emerged adults (LR *χ*^2^_6_ = 9.15, *P* = 0.165) (Fig. 1b). However, posthoc tests indicated that TiO_2_NPs reduced larva-to-adult survival and egg-to-adult survival (or the number of emerged adults) by 10.8% and 15.0%, respectively, compared to the control (larva-to-adult survival, *P* = 0.011; egg-to-adult survival, *P* = 0.034; emerged adults, *P* = 0.021, Fig. 1b).

#### Treatment on larvae of *C. maculatus*

There was no difference in larva-to-adult survival among the NP elements and the control (LR *χ*^2^_6_ = 2.64, *P* = 0.852, Fig. 2b), with one outlier in the control group excluded from the analysis.

### Egg sizes of two *Callosobruchus* species

Egg mass was different between the two species (*F*_1,90_ = 107.7, *P* < 0.001), with *C. chinensis* eggs being 22.9% smaller (0.0212 ± 0.00044 mm^3^, mean ± SE, *n* = 50) than *C. maculatus* eggs (0.0275 ± 0.00037 mm^3^, *n* = 45)*.* Parental pair ID had a significant effect (*F*_3,90_ = 3.8, *P* = 0.013).

## Discussion

We compared the entomotoxic efficacy of the six types of nanoparticles (NPs) biosynthesized using the fungal extract from *F. solani* on the immature stages of *C. chinensis* and *C. maculatus*. Our results showed that susceptibility to biosynthesized NPs varied by species and developmental stage. The eggs of both species were more susceptible than the last-instar larvae, which were protected by the seed coat. This suggests that direct contact with nanopesticides is crucial for controlling pest populations. When beetle eggs were treated, SeNPs and TiO_2_NPs reduced egg hatchability in *C. chinensis*, and larval-to-adult survival in *C. maculatus*, leading to a reduction in the egg-to-adult survival by SeNPs in *C. chinensis* and by TiO_2_NPs in *C. maculatus*. Since the eggs of *C. chinensis* were 23% smaller than those of *C. maculatus* (in line with^53^), the surface area to volume ratio was higher, resulting in greater exposure of *C. chinensis* eggs to NPs. This could explain the difference in acute NP efficacy against eggs between the two species. In contrast, when beans containing beetle larvae were treated with NPs, no effect was observed. Since eggs and larvae are similarly more vulnerable than adults when NPs are applied directly^52^, the apparent resistance of the larvae against the NPs is possibly due to the indirect method of application via the seed coat. The biosynthesized NPs, particularly SeNPs and TiO_2_NPs, showed the potential to control the major stored bean pests when applied to eggs attached to the surface of seed coat but not when applied to larvae inhabiting in seeds.

This is one of the early demonstrations of the insecticidal effects of SeNPs^14,26^. Se-based organic molecules can produce reactive oxygen species (ROS) and trigger apoptosis or autophagy of cancer cells^21^. Sodium selenite induces dose-dependent mortality and dose-dependent accumulation of selenium in the Malpighian tubules of the mealworm beetle *Tenebrio molitor* but not in the digestive and reproductive organs^54^, while SeNPs synthesized with plant extracts cause damages on larval cellular components of a mosquito, such as nucleus, lumen, and gut epithelial cells^55^. However, the mechanism of the effect of SeNPs still remains largely unexplored^56^. Similarly, TiO_2_NPs can generate ROSs^27^. The efficacy of TiO_2_NPs has been compared to other NPs: the efficacy of TiO_2_NPs is higher than AgNPs (on *Spodoptera litura* larvae^57^) and ZnONPs (on *Sitophilus oryzae* adults^58^), in support of our results, regardless of differences in species tested. TiO_2_NPs synthesized with plant extracts increase the activity of detoxification enzymes and cause histopathological change in the midgut of *S. litura* and a mosquito^59^.

Although SeNPs have been synthesized using fungi^26,39,60^ and plant extracts^55^, their efficacy has not been tested on stored product pests before. Our study is the first to demonstrate the entomotoxic efficacy of biosynthesized SeNPs and TiO_2_NPs on stored product pests, and the first to test the efficacy of *Fusarium*-synthesized NPs on insects. However, the influence of dose dependency remains to be tested (e.g.^14,54,59,61,62^), as low doses of NPs can enhance insect performance (^63^, Miksanek et al. under review).

## Conclusion

Our results suggest that the direct applications of SeNPs and TiO_2_NPs to eggs are most effective to control the stored bean pests, *C. chinensis* (18.1% reduction in egg-to-adult survival compared to the control) and *C. maculatus* (15.0% reduction in egg-to-adult survival compared to the control), respectively. Quantitative studies regarding impact on optimal dosages for effective control of multiple species of pests with minimum side-effects on crops^18,50,64,65^, and comparison with their conventional analogues are imperative in the future. Our study cautions that the efficacy of nanopesticides in controlling pests depends on the target developmental stages; direct application of nanopesticides to the highly vulnerable early immature stages of pests is recommended for optimal control.

## Materials and methods

### Fungal culture

The fungal culture used for synthesizing different NPs in this study was isolated from a soil sample collected from the pots used for the experimental studies at the Laboratory of Insect Natural Enemies, Faculty of Agriculture, Kyushu University, using the direct plating method^66^. The isolated strain was morphologically differentiated using the classification system by Smith and Onion^67^. Molecular classification was performed using the method described by Henry et al.^68^, which is detailed in the following section.

### Molecular identification of fungi

The fungal isolate was identified based on the ITS rDNA sequence amplified with the primers ITS1 and ITS4^68^. First, the DNA was extracted by freezing and thawing a small sample of the fungal colony dissolved in TE buffer. The PCR was conducted with an annealing temperature at 53 °C using KOD One (Toyobo, Tokyo, Japan), following the manufacturer’s protocol. The PCR product was purified and subjected to Sanger sequencing. The sequence data were searched for matches in the database nr using BLASTn (NCBI, MD, USA). The fungal isolate was identified with 100% certainty as *Fusarium solani* (Hypocreales: Nectriaceae) through morphological differentiation and genotypical identification based on the ITS sequence.

### Biosynthesis of nanoparticles using fungi

To prepare the biomass for biosynthesis of metal and non-metal NPs, fungal culture was grown aerobically in liquid media consisting of 3.0 g malt extract, 10.0 g glucose, 2.0 g yeast extract, 5.0 g peptone, 20.0 g agar–agar and 1.0 L distilled water, with pH adjusted to 6.2 as per^69^. The fungal culture was filtered aseptically and incubated in sterilized deionized water for 72 h under aerobic conditions.

Silver (Ag) NPs were synthesized by adding 500 mg L^−1^ of AgNO_3_ solution to the cell-free water extract of the fungal isolate. The reduction of Ag ions to AgNPs was confirmed by the color transformation of the mixture to brown^70^ (Supplementary Fig. S1a). Copper oxide (CuO) NPs were synthesized by adding 500 mg L^−1^ of Cu*II*SO_4_ solution to the cell-free water extract of the fungal isolate. The reduction of Cu ions to CuONPs was confirmed by the color transformation of the mixture to blue-green (Fig. S1b). Selenium (Se) NPs were synthesized by adding 500 mg L^−1^ of Na_2_SeO_3_ solution to the cell-free water extract of the fungal isolate. The reduction of Se ions to SeNPs was confirmed by the color transformation of the mixture to red (Fig. S1c). Silicon dioxide or silica (SiO_2_) NPs were synthesized by adding 500 mg L^−1^ of SiO_2_ solution to the cell-free water extract of the fungal isolate. No color transformation of the mixture was observed (Fig. S1d). Titanium dioxide (TiO_2_) NPs were synthesized by adding 500 mg L^−1^ of TiO_2_ solution to the cell-free water extract of the fungal isolate. The reduction of Ti ions to TiO_2_NPs was confirmed by the color transformation of the mixture to a deep white colloidal solution (Fig. S1e). Zinc oxide (ZnO) NPs were synthesized by adding 500 mg L^−1^ of ZnSO_4_.7H_2_O solution to the cell-free water extract of the fungal isolate. No color transformation of the mixture was observed (Fig. S1f). The characterization of the resulting NPs was carried out using transmission electron microscopy (TEM) and energy-dispersive X-ray spectroscopy (EDX) as described below.

### Characterization of nanoparticles

The size and shape of the different NPs synthesized using the fungal isolate were determined using TEM (Philips Tecnai-G2 20, Japan). To prepare TEM samples, a drop of well-dispersed NP solution was placed onto conventional carbon-coated copper TEM grids (150 μm meshes, Plano GmbH, Germany), and the drop was allowed to dry overnight in a desiccator before imaging. Three TEM images of each sample were obtained for morphological analysis and particle size using an accelerating voltage of 200 kV. To analyze the elemental chemical composition of the NPs, the EDX spectra were examined coupled with the TEM (Tecnai-G2 20).

The six types of NPs produced by the *F. solani* isolate were characterized using TEM and EDX as follows (EDX: Supplementary Fig. S2): The spherical AgNPs produced by this fungal extract had a diameter of 15.3 ± 0.2 nm (mean ± SE). The spherical CuONPs produced had a diameter of 11.7 ± 0.3 nm and the spherical SeNPs produced had a diameter of 20.0 ± 0.1 nm. The size of the amorphous SiO_2_NPs produced was 32.9 ± 2.6 × 75.1 ± 8.9 nm. Finally, the spherical TiO_2_NPs had a diameter of 15.4 ± 0.2 nm and the ZnONPs had a diameter of 8.1 ± 0.5 nm.

### Efficacy test on pest bean beetles

To test the efficacy of the above-mentioned fungus-synthesized NPs against immature stages, egg (attached to the surface of seeds) and larva (feeding seeds internally) of stored product pests, we used two species of stored bean pest beetles: *Callosobruchus chinensis* (Coleoptera: Chrysomelidae: Bruchinae) strain jC, which has been maintained on dried azuki beans [*Vigna angularis* var. *angularis* (Fabaceae), purchased from Daiwa grain, Obihiro, Japan] under a laboratory condition at 30 °C for over 70 years^10,71^. The other species *Callosobruchus maculatus* strain tQ has also been maintained on azuki beans under the same laboratory condition as *C. chinensis* for over 30 years^72^. Each of the biosynthesized NPs was directly applied to the seed coat of azuki beans that were either with beetle eggs on the surface or infested by beetle larvae. Each treatment was replicated for 9 times, except for the controls for *C. chinensis* (11 times for egg treatment and 10 times for larval treatment) and for the control for *C. maculatus* (11 times for egg treatment) at 30 °C, 60% r.h. and 16L:8D.

#### Direct application of nanoparticles to eggs

Eggs were deposited for 2 h on azuki beans by females that emerged within 24 h. Beans with 1–2 eggs were chosen. Seven to eight beans with a total of 10 eggs of 24 h old were introduced to a petri dish (6 cm diameter) and 20 μL (10 μg) of the biosynthesized NP solution or distilled deionized water was applied with a micropipette, and the dish was gently agitated to coat the bean and egg surface with the NPs. After eight days from application, hatched eggs were counted. Emerged adults were counted after 37 days from egg deposition to rear the treated eggs into adults, via larvae and pupae. A total of 650 eggs for *C. chinensis* and 650 eggs for *C. maculatus* were used for this experiment.

#### Application of nanoparticles to larvae

Twenty μl (10 μg) of the biosynthesized NP solution or distilled deionized water was applied to seven to eight azuki beans infested by a total of 10 fourth instar larvae (14 days old) at a density of 1–2 larvae/bean in a petri dish (6 cm diameter). The dishes were gently agitated. After 23 days of rearing the treated larvae into adults under the same environmental conditions (i.e., 37 days from egg deposition), emerged adults were counted. A total of 640 larvae for *C. chinensis* and 620 larvae for *C. maculatus* were used for this experiment.

### Egg sizes of two *Callosobruchus* species

To explain the possible efficacy difference on eggs between the two species, we estimated the egg mass of the two species based on the length and width of eggs, using the equation by Yanagi and Tuda^73^. The length and width of hatched eggs laid by each female of two (*C. chinensis*) or three (*C. maculatus*) pairs on 20 untreated azuki beans in petri dishes (6 cm in diameter) were measured to the precision of 0.001 mm with a microscope (H-5500, Keyence, Osaka, Japan).

All methods were carried out in accordance with relevant institutional, national, and international guidelines and legislation.

### Statistics

We tested the effect of NP element on the life history traits of each species studied: Logistic regression analyses were performed on the survival of eggs (that is, egg hatchability), larva to adult, and egg to adult of each beetle species, with NPs or water as an explanatory variable, followed by posthoc comparisons with the control. Egg mass was tested with a general linear model, with NPs or water treatment and parental pair ID nested within treatment as explanatory variables, confirming the normality of the residual errors. All statistical tests were performed using JMP 14.2.0.

## Supplementary Information

Supplementary Figures.

## Data Availability

The datasets associated with the current study are available from the primary corresponding author (M. Tuda: tuda@grt.kyushu-u.ac.jp) upon reasonable request.
