# Supplementary material for: Entomotoxic efficacy of fungus-synthesized nanoparticles against immature stages of stored bean pests
Source: Sci Rep. 2023 May 25;13:8508. doi: 10.1038/s41598-023-35697-1 (PMC10212994; doi:10.1038/s41598-023-35697-1)
Supplement: Supplementary file 1 — Supplementary Figures. [file 41598_2023_35697_MOESM1_ESM.docx]

**Supplementary Materials**

**Entomotoxic efficacy of fungus-synthesized nanoparticles against immature stages of stored bean pests**

Eman Ahmed Mohamed Helmy^1,2,^*, Phyu Phyu San^2,3^, Yao Zhuo Zhang^2^, Charles Adarkwah^2,4,5^, Midori Tuda^2,^*

^1^ The Regional Centre for Mycology and Biotechnology (RCMB), Al-Azhar University, Cairo, Egypt

^2^Laboratory of Insect Natural Enemies, Institute of Biological Control, Faculty of Agriculture, Kyushu University, Fukuoka 819-0395, Japan

^3^ Department of Entomology and Zoology, Yezin Agricultural University, Naypyitaw, Myanmar

^4^ Department of Horticulture and Crop Production, School of Agriculture and Technology, Dormaa-Ahenkro Campus, University of Energy and Natural Resources, PO Box 214, Sunyani, Ghana

^5^Division Urban Plant Ecophysiology, Faculty Life Sciences, Humboldt-University of Berlin, Lentzeallee 55/57, 14195 Berlin, Germany

**Corresponding authors**: E.A.M. Helmy; M. Tuda

emanhelmo@yahoo.com; tuda@grt.kyushu-u.ac.jp

**Supplementary Figure S1** Synthesis of (a) Ag nanoparticles, (b) CuO nanoparticles, (c) Se nanoparticles, (d) SiO_2_ nanoparticles, (e) TiO_2_ nanoparticles and (f) ZnO nanoparticles, using the extract from the fungus *Fusarium solani*. Left bottles: before the synthesis of nanoparticles. Right bottles: after the synthesis.

|  | **Supplementary Figure S2** Energy-dispersive X-ray spectrum (EDX) of (a) Ag nanoparticles, (b) CuO nanoparticles, (c) Se nanoparticles, (d) SiO_2_ nanoparticles, (e) TiO_2_ nanoparticles and (f) ZnO nanoparticles, all synthesized using the extract from the fungus *Fusarium solani.* |
| --- | --- |
